# Relationship between malnutrition and coronary microvascular dysfunction in patients with nonischemic dilated cardiomyopathy

**DOI:** 10.3906/sag-2003-239

**Published:** 2020-12-17

**Authors:** Şeref KUL, Tolga Sinan GÜVENÇ, Mustafa ÇALIŞKAN

**Affiliations:** 1 Department of Cardiology, Medicine of Faculty, Medeniyet University Göztepe Research and Training Hospital, İstanbul Turkey; 2 Department of Cardiology, VM Medical Park Pendik Hospital, İstanbul Turkey

**Keywords:** Transthoracic echocardiography, nonischemic dilated cardiomyopathy, coronary flow reserve, malnutrition, inflammation

## Abstract

**Background/ aim:**

Malnutrition is common in patients with nonischemic dilated cardiomyopathy (DCM), especially in the end stages of the disease where heart failure symptoms predominate. Malnutrition has been associated with atherosclerosis in patients with chronic kidney disease, but it is unknown whether a similar relationship exists between malnutrition and coronary microvascular dysfunction (CMD). In the present study, we aimed to analyse whether indices of malnutrition were associated with coronary flow reserve (CFR) in patients with DCM.

**Materials and methods:**

A total of 33 cases who were prospectively followed up with by institutional DCM registry were found eligible for inclusion. Coronary flow reserve was measured with transthoracic echocardiography from the left anterior descending artery. The study sample was divided into 2 groups using a CFR cut-off value of 2.0. Geriatric nutritional index (GNI), prognostic nutritional index (PNI), and C-reactive protein/albumin ratio (CAR) were calculated.

**Results:**

A total of 17 out of 33 cases (51.5%) had a low (<2.0) CFR. Both GNI and PNI were similar between the 2 groups, but the inflammatory–nutritional parameter CAR was significantly higher in those with a low CFR (1.18 ± 0.64 vs. 0.54 ± 0.28, P < 0.001). CA remained an independent predictor of CFR on multivariate regression (β = 0.65, P < 0.001) after adjustment for demographic (age, sex, body mass index), nutritional (GNI, PNI, albumin), and inflammatory (C-reactive protein) parameters. For a cut-off value of 0.80, CAR had a sensitivity of 85.7% and specificity of 73.6% to predict a CFR <2.0 (AUC: 0.835, 95%CI: 0.693–0.976, P = 0.001).

**Conclusions:**

Our findings indicate that not malnutrition per se but a combination of inflammation activation and malnutrition is predictive of CMD in patients with DCM.

## 1. Introduction

Nonischemic dilated cardiomyopathy (DCM) is the final consequence of various pathologic processes that cause myocardial damage and is a common cause of heart failure (HF) syndrome. By definition, DCM excludes significant epicardial coronary disease or previous myocardial infarction as the cause of initial myocardial insult, but a reduced coronary flow reserve (CFR) is observed in a fraction of DCM patients [1,2]. This reduction in coronary flow reserve (CFR) is explained by coronary microvascular dysfunction (CMD), which is secondary to structural alterations, elevated left ventricular end-diastolic pressure externally compressing intramyocardial arterioles and capillaries, and endothelial dysfunction (ED) due to reduced bioavailability of nitric oxide and formation of free radicals [3]. Reduced CFR is associated with worsening left ventricular function, disease progression, and an overall worse prognosis in DCM patients [4].

Patients with HF syndrome, including those with DCM, are in an inflammatory–catabolic state, which can manifest itself as cardiac wasting at the latter stages of the disease where HF syndrome predominates [5,6]. Malnutrition is a common finding, seen in up to 50% of patients with HF, and is related with increased morbidity and mortality [7]. Given the strong association between dietary patterns and micronutrients with endothelial function, it is plausible to consider malnutrition as a possible mechanism aggravating CMD [8]. In our experience, CMD is related to abnormal liver enzymes and metabolism end-products such as uric acid in DCM patients, suggesting that abnormalities in metabolism are linked to abnormal microvascular function [9,10]. Data in patients with chronic kidney disease (CKD) undergoing dialysis suggest such an association, which is termed malnutrition, inflammation, and atherosclerosis (MIA) syndrome [11,12]. The effects of inflammation and malnutrition in other patients, such as those with DCM, as well as the impact on microvascular function, are practically unknown due to a lack of relevant data.

In the present study, we aimed to study the relationship between CFR and nutritional indices, including geriatric nutritional index (GNI), prognostic nutritional index (PNI), inflammatory–nutritional index, and C-reactive protein (CRP) to albumin ratio (CAR) in a pilot study in order to understand the effects of nutrition on CMD and to investigate whether these indices could be useful for predicting CFR in patients with DCM.

## 2. Materials and methods

For the present study, we used data from an institutional registry that prospectively follows up with patients with DCM. Patients that were older than 18 years old and had at least 2 echocardiographic examinations that were done 6 months apart and showed a left ventricular (LV) ejection fraction of ≤50% and evidence for LV dilation (LV end-diastolic volume greater than 2 SD from an upper reference limit corrected for age and sex) were screened for inclusion to the present work. All patients included in the present study underwent coronary angiography as part of their initial DCM work-up, and patients with any evidence of atherosclerotic coronary artery disease on this angiography or a history of myocardial infarction were excluded regardless of the significance of underlying coronary artery disease. Other exclusion criteria included the following: cardiac conditions with known effects on CFR, such as other types of cardiomyopathies; a medical history of systemic diseases with known effects on microvascular function, such as diabetes, hypertension, thyroid diseases, or systemic rheumatologic disorders; other organ failures that may affect CFR, such as stage 3 or more advanced chronic kidney disease or moderate to severe liver impairment; and those with conditions that may affect measurement of CFR, such as persistent atrial arrhythmias or very frequent extrasystoles. Finally, patients that were smokers and those with a history of substance abuse (including alcohol) were also excluded given the close relationship between these habits and CFR. A total of 33 cases were found eligible based on these criteria and were subsequently enrolled. All patients included to the present study had New York Heart Association (NYHA) class 2–3 HF and received guideline-directed medical treatment at the maximum doses tolerated. Demographic, anthropometric, clinical, laboratory, and echocardiographic data were collected from registry data using paper charts or electronic databases.

The present study was conducted according to the principles of the 1975 Declaration of Helsinki. An ethics approval was obtained from a local ethics committee before starting the study. All patients gave their written consent before inclusion to the registry, but no additional consent was sought for the present study.

### 2.1. Echocardiographic assessment of coronary flow reserve

Detailed methodology for the echocardiographic measurement of CFR has been presented before [13]. In brief, all measurements were done from the left anterior descending artery (LAD) by visualizing the LAD from a modified 2- or 4-chamber view while the patient was in the left lateral position. Coronary blood flow velocities were measured using pulsed wave Doppler at rest immediately after dipyridamole infusion (0.56 mg/kg over 4 min). The 5 highest diastolic velocities recorded during the test were averaged to obtain the diastolic peak flow velocities (DPFV) at baseline, during maximal dipyridamole infusion, and 3 min after cessation of dipyridamole. All echocardiographic examinations were performed by an investigator experienced in echocardiography and echocardiographic assessment of CFR (Dr. MC) using an ultrasound platform (Philips EPIQ 7G, Bothell, WA, USA) equipped with a broadband S5-1 transducer (transmission frequency: 1.7 MHz; receiver frequency: 3.4 MHz). CFR was defined as the ratio of hyperemic diastolic peak velocity to baseline diastolic peak velocity, and a CFR ≥ 2.0 was considered normal.

### 2.2. Calculation of nutritional indices

Geriatric nutritional index was calculated using serum albumin, height, and body weight using the following formula [14]: GNI = [14.89 × albumin (g/dL)] + [41.7 × (body weight / ideal body weight)]; where ideal body mass index (BMI) was assumed as 22 kg/m2 and ideal body weight was calculated using ideal BMI and actual height.

Prognostic nutritional index was calculated using serum albumin and absolute lymphocyte count in the peripheral blood using the following formula [15]: 10 × serum albumin value (g/dL) + 0.005 × absolute lymphocyte count (/mm3).

The inflammatory–nutritional parameter CAR was calculated by simply dividing CRP (mg/dL) by serum albumin [16].

## 3. Statistical analyses

All statistical analyses were performed using JASP (JASP Team, 2019; Version 0.10.2 for Windows) and SPSS 17.0 (IBM Inc., Armonk, NY, USA) statistical software. Patients were divided into 2 subgroups according to CFR. Continuous variables were given as mean ± SD, while categorical variables were presented as percentages. For continuous variables, Shapiro–Wilk and Levene’s tests were used to analyse distribution patterns and equality of variances in these subgroups, respectively. Student’s t-test, with Welch correction if the variances were not equal, was used for variables with a normal distribution. For parameters without a normal distribution pattern, the Mann–Whitney U test was used. Correlation analyses were done with the Pearson test for parameters with a normal distribution, and Spearman’s rho was used for parameters with a severely skewed distribution. For categorical variables, ki2 or Fisher’s exact tests were used depending on expected cell counts. Receiver–operator characteristic (ROC) curves were drawn to determine accuracy and optimal cut-off for variables that had the highest likelihood for predicting a CFR < 2.0. For nutritional parameters that offered the highest accuracy for predicting CFR, cut-off values were determined using ROC curves; these cut-off values were used to convert scalar nutritional indices into binomial parameters. A multivariate logistic regression model with forward likelihood selection criteria was built to adjust the effects of confounders on the tested variables. Categorical variables were introduced to the multivariate liner regression model using dummy parameter coding. This model was bootstrapped 5000 times to improve overall model accuracy. Finally, a logistic regression model was built using binomial parameters to study whether cut-off values found on ROC curves offered adequate predictive capability to determine a low (<2.0) CFR in DCM patients. A P value of <0.05 was accepted as the limit for statistical significance.

## 4. Results

Mean age of the study population was 55.64 ± 11.24 and 23 (69.7%) of the patients were male, with a mean BMI of 27.62 ± 4.38 kg/m2. Mean ejection fraction and CFR were 0.37 ± 0.09 and 2.05 ± 0.39, respectively, for the whole study group. Sixteen patients had a CFR ≥ 2.0 (48.5%), while the remaining 17 patients (51.5%) had a CFR < 2.0. Mean hemoglobin, glucose, total cholesterol, triglycerides, and albumin levels were 14.18 ± 0.96 g/dL, 99.03 ± 8.64 mg/dL, 172.55 ± 36.92 mg/dL, 141.58 ± 57.87 mg/dL, and 3.95 ± 0.45 g/dL, respectively. Mean GNI and PNI were 111.41 ± 12.32 and 49.15 ± 6.87 in the study population, while median CAR was 0.87 ± 0.59.

### 4.1. Coronary flow reserve subgroups

Demographic, clinical, laboratory, and echocardiographic data for patients with CFR <2.0 and ≥2.0 are summarized in Table 1. There were no significant differences between groups for age, sex, or BMI, but there was a trend toward higher systolic and diastolic blood pressure in those with a low CFR. On echocardiography, left ventricular ejection fraction was lower (P = 0.06) and left ventricular filling pressure was significantly higher (P = 0.001) in the subgroup of patients with CFR <2.0. Albumin, total cholesterol, and triglyceride levels were all lower in those with a CFR < 2.0, although none were statistically significant. In contrast, CRP was significantly higher in patients with a CFR <2.0. For nutritional variables, only CAR showed a significant elevation in the subgroup of patients with a CFR <2.0, while no statistically significant differences were observed for other parameters (Table 1).

**Table 1:** Demographic, clinical, laboratory, and echocardiographic characteristics of dilated cardiomyopathy patients grouped according to coronary flow reserve (CFR). Note that patients with a low CFR had similar geriatric and prognostic nutritional indices as compared to those with a normal CFR, but CRP to albumin ratio was significantly higher in the former group. Comparisons with a P value below 0.05 were written in bold. CFR, coronary flow reserve; DCM, dilated cardiomyopathy; BP, blood pressure; CRP, C-reactive protein; LV, left ventricle; BMI, body mass index.

Parameter	DCM patients with CFR ≥ 2.0	DCM patients with CFR < 2.0	P
Demographic and anthropometric variables			
Age (years)	56.1 ± 9.7	55.2 ± 12.9	0.81
Sex (male%)	12 (75.0%)	11 (64.7%)	0.71
Height (cm)	168.7 ± 9.1	165.5 ± 9.9	0.35
Weight (kg)	76.8 ± 14.1	78.3 ± 17.1	0.79
BMI (kg/m2)	27.22 ± 3.81	27.99 ± 4.97	0.63
Clinical variables			
Systolic BP (mmHg)	116.4 ± 8.1	124.7 ± 14.9	0.06
Diastolic BP (mmHg)	74.3 ± 4.5	79.2 ± 8.0	0.07
Heart rate (beats/min)	73.1 ± 9.8	71.0 ± 15.0	0.65
Laboratory variables			
White blood cell count (x103)	8.03 ± 2.28	8.62 ± 1.68	0.40
Hemoglobin (g/dL)	14.24 ± 0.94	14.12 ± 1.00	0.97
Lymphocyte count (×103)	1.87 ± 0.89	1.97 ± 1.03	0.66
CRP (mg/dL)	2.17 ± 1.00	4.52 ± 2.42	0.001
Blood glucose (mg/dL)	99.93 ± 9.40	98.25 ± 8.15	0.60
Blood urea nitrogen (mg/dL)	14.60 ± 2.97	19.25 ± 8.82	0.27
Creatinine (mg/dL)	0.97 ± 0.24	1.12 ± 0.32	0.20
Albumin (g/dL)	4.04 ± 0.48	3.86 ± 0.42	0.26
Total cholesterol (mg/dL)	177.81 ± 39.69	167.59 ± 34.56	0.44
Triglycerides (mg/dL)	146.19 ± 60.51	137.24 ± 56.77	0.66
Sodium (meq/L)	140.38 ± 1.89	139.60 ± 2.26	0.33
Echocardiographic variables			
LV end-diastolic volume (mL)	178.20 ± 47.92	219.35 ± 74.86	0.10
LV end-systolic volume (mL)	110.53 ± 47.37	146.76 ± 52.54	0.04
LV ejection fraction (%)	39.71 ± 9.71	33.59 ± 7.67	0.06
Mean E/e’ ratio	6.53 ± 2.05	13.52 ± 8.47	0.001
Nutritional indices			
Geriatric nutritional index	111.26 ± 11.17	111.55 ± 13.66	0.95
Prognostic nutritional index	49.81 ± 7.16	48.52 ± 6.74	0.66
CRP: albumin ratio	0.54 ± 0.28	1.18 ± 0.64	<0.001
Flow indices			
Basal flow velocity (m/s)	25.44 ± 5.46	29.12 ± 8.81	0.25
Hyperaemic flow velocity (m/s)	59.88 ± 14.76	51.00 ± 14.20	0.10
Coronary flow reserve	2.36 ± 0.31	1.76 ± 0.15	<0.001

### 4.2. Diagnostic accuracy and optimal cut-off value for C-reactive protein/albumin ratio to determine coronary flow reserve

**Figure 1:**
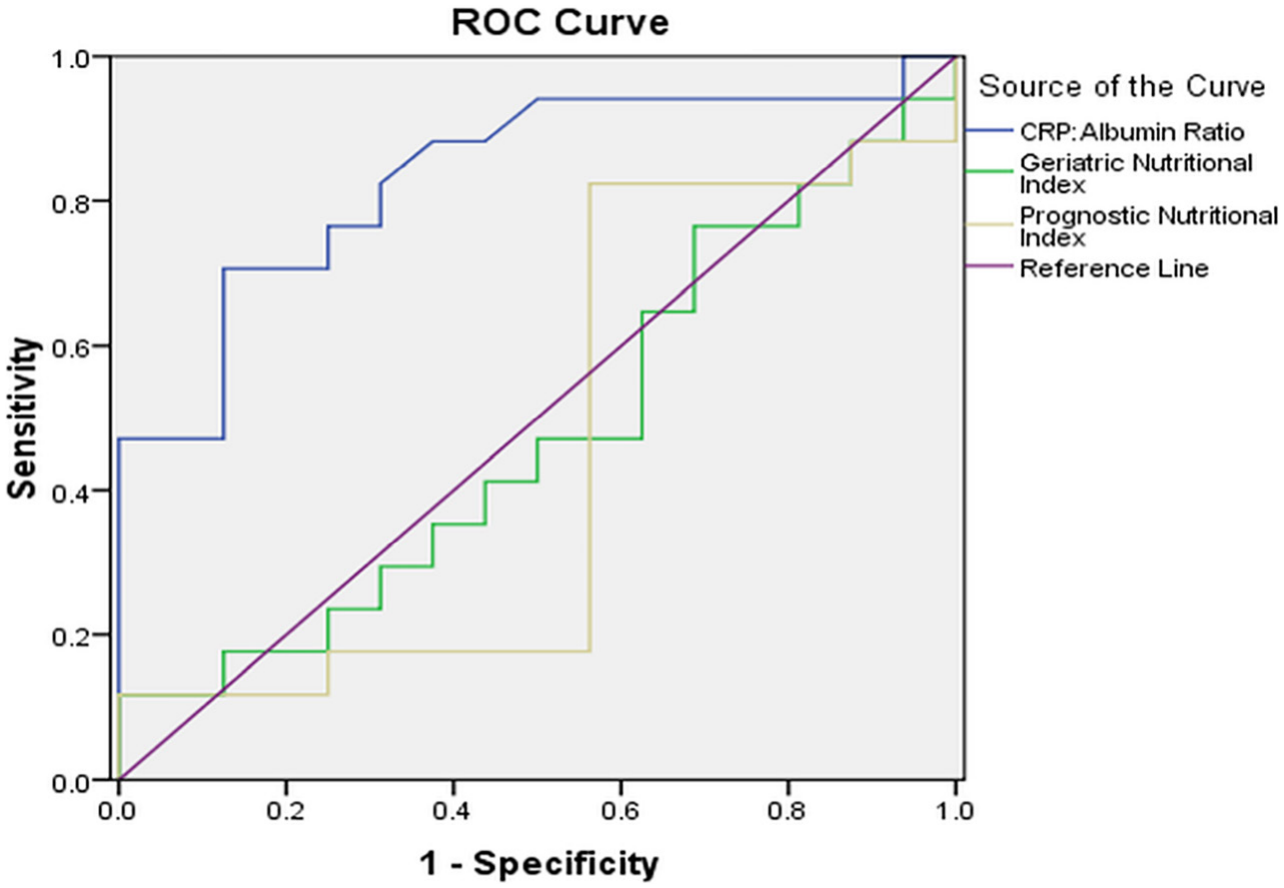
Receiver-operator characteristic (ROC) curves showing accuracy of nutritional indices to predict a coronary flow reserve <2.0 in patients with nonischemic dilated cardiomyopathy. Of all indices studied, only C-reactive protein to albumin ratio (blue line) had a statistically significant c-statistic (AUC 0.835, P = 0.001) to predict a low coronary flow reserve. See text for details. CRP, C-reactive protein.

When all nutritional parameters were analysed for their accuracy to determine CFR in DCM patients, only CAR was found to have a statistically significant area under curve (AUC) to determine a CFR of less than 2.0 (AUC: 0.835, 95% CI: 0.693–0.976, P = 0.001). In contrast, both GNI (AUC: 0.522, P = 0.83) and PNI (AUC: 0.548, P = 0.64) did not have a statistically significant association with CFR (Figure 1). For a cut-off value of 0.80, CAR had a sensitivity of 85.7%, specificity of 73.6%, a positive predictive value of 70.5%, and a negative predictive value of 87.5%.

### 4.3. Adjustment for confounding factors

On univariate analysis, CAR had a moderate to strong correlation with CFR with a correlation coefficient of –0.73 (P <0.001). A multivariate model was used to adjust that association between CAR and CFR for demographic and anthropometric variables (age, sex, and BMI) and other nutritional indices (GNI and PNI). Additionally, this model was adjusted for CRP and albumin to understand whether CAR would have a predictive value independent of its constituent variables. CAR remained as the only independent parameter that was associated with CFR following multivariate adjustment; this finding remained the same after bootstrapping the model (Table 2). A similar finding was obtained when CAR was included in the logistic regression model with the same covariates, where CAR was the sole parameter that determined a CFR <2.0 with an odds ratio of 14.30 (95% CI: 2.30–88.8, P = 0.004).

**Table 2:** Univariate and multivariate predictors of coronary flow reserve in patients with dilated cardiomyopathy. Note that parameters other than C-reactive protein to albumin ratio were included to the model for adjustment. The results remained valid after bootstrapping the model for 5000 iterations. CRP, C-reactive protein; BMI, body mass index; GNI, Geriatric nutritional index; PNI, prognostic nutritional index.

Parameter	Univariate correlation		Multivariate regression	
	r	P	β	P
CRP: albumin ratio	–0.73	<0.001	–0.65	<0.001
CRP (adjustment)	–0.70	<0.001		
Albumin (adjustment)	0.18	0.32		
Age (adjustment)	0.09	0.63		
Sex (adjustment)	–0.01	0.97		
BMI (adjustment)	–0.01	0.98		
GNI (adjustment)	0.03	0.89		
PNI (adjustment)	0.19	0.29		

### 4.4. Correlation of C-reactive protein/albumin ratio with other echocardiographic variables

**Figure 2:**
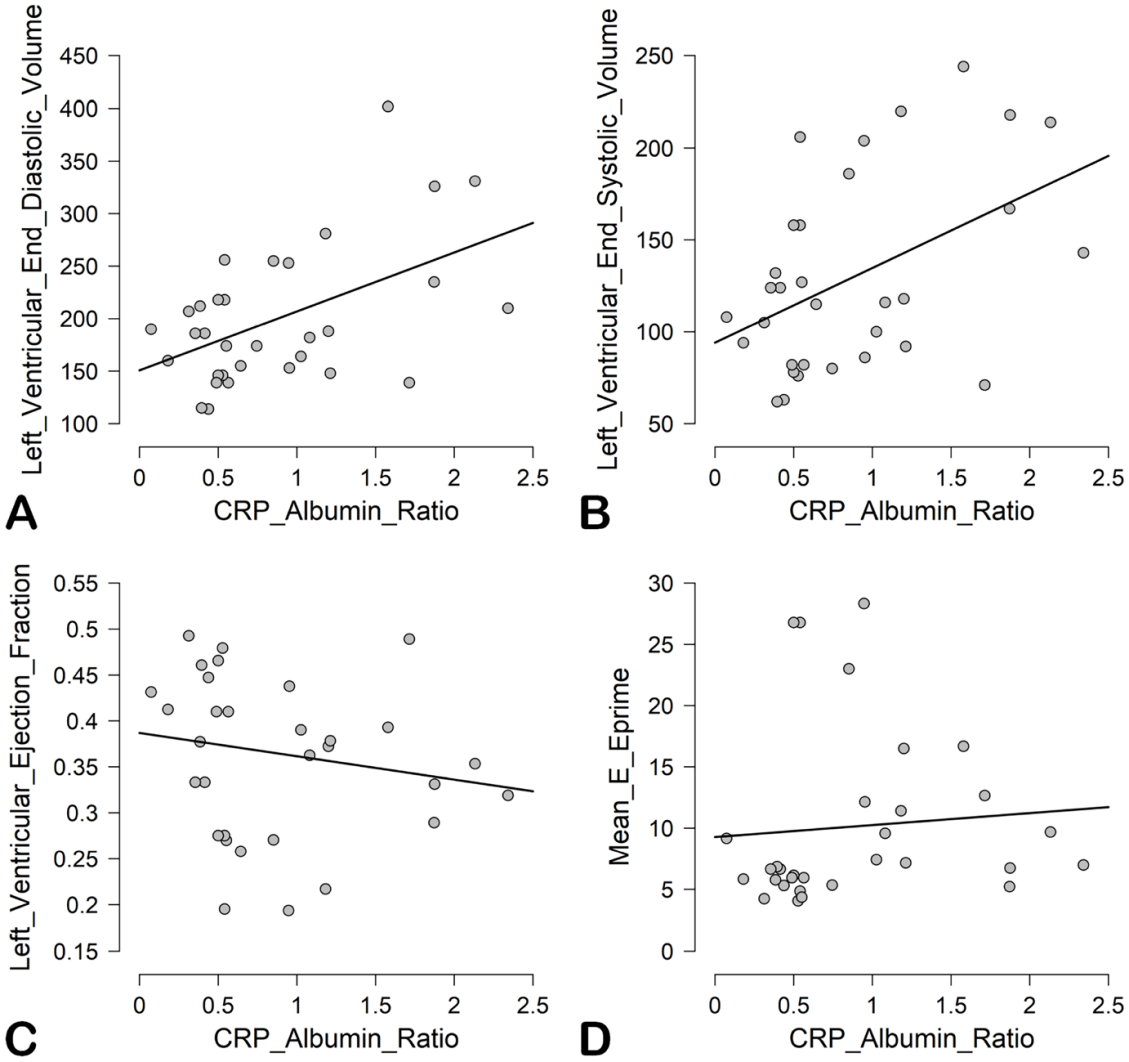
Scatter plots showing correlation between C-reactive protein to albumin ratio with various echocardiographic measurements. C-reactive protein to albumin ratio had statistically significant correlations with left ventricular end-diastolic pressure and endsystolic pressure (panels A and B), but not with left ventricular ejection fraction or mean E/e’ ratio (panels C and D), the latter being an echocardiographic surrogate for left ventricular filling pressure. See text for details. CRP, C-reactive protein.

C-reactive protein/albumin ratio had statistically significant correlations with left ventricular end-diastolic volume (r = 0.51, P = <0.001) and LV systolic volume (r = 0.46, P = 0.01). In contrast, no such associations were observed between CAR and left ventricular ejection fraction (r = –0.17, P = 0.36) or between CAR and mean E/e’ ratio (r = 0.08, P = 0.66), the latter being an echocardiographic marker of left ventricular filling pressure (Figure 2).

## 5. Discussion

In the present study, we sought to understand the relationship between nutritional indices and CFR in patients with nonischemic DCM. Key takeaways from the present study are as follows: *i)* Of all nutritional indices studied, only the inflammatory–nutritional index CAR has shown an association with CFR, while sole nutritional indices such as GNI or PNI did not have such a relationship with CFR; *ii)* This association between CAR and CFR was independent of confounders, including parameters that constituted CAR, namely CRP and albumin; *iii)* Using a cut-off value of 0.80, CAR offers an accuracy of 78.8% for correct estimation of a CFR <2.0; and iv) CAR showed a significant correlation with LV dimensions, but not with LV ejection fraction or filling pressure.

### 5.1. Inflammation, malnutrition, and coronary flow reserve in patients with nonischemic dilated cardiomyopathy

Coronary microvascular flow is affected by several factors, including endothelial function, metabolic factors, capillary density in myocardium, and external compression [3]. By affecting NO metabolism and bioavailability in vascular endothelium, inflammation has well-known effects on endothelial function; both acute and chronic inflammation lead to ED and hence CMD. Endothelial and coronary microvascular functions are also affected by dietary patterns, where unhealthy dietary habits were associated with ED and vascular aging [8]. Micronutrients such as dietary electrolytes (potassium, sodium, magnesium) exert a powerful modifier effect on vascular function; consumption of specific foods (whey protein, legumes, olive oil, whole grains, and similar “healthy” foods) improves endothelial and vascular function by reducing oxidative stress and inflammation [17–21]. Malnutrition is frequently observed with inflammation and atherosclerosis as a triad in patients with chronic kidney disease who undergo peritoneal dialysis or hemodialysis, thus suggesting a possible causal role for malnutrition in the development and progression of atherosclerosis [11,12]. While there is no solid evidence linking malnutrition with CMD, such an association is likely, given that ED and CMD are precursor events for the development of atherosclerotic lesions [22]. A similar mechanism could be operational in DCM, where absorption and utilization of nutrients are impaired and the overall metabolism shifts to a catabolic state, thus leading to malnutrition.

Despite these theoretical considerations, the present findings do not support an independent association between indices of nutrition (GNI and PNI) and CFR. Both indices are well-established markers of inflammation that are predictors of mortality in patients with HF in various clinical settings and, as such, the most straightforward explanation of this finding is a true lack of association between nutrition and CFR in DCM [23,24]. That said, mean GNI and PNI in the study group (111.4 and 49.2), as well as in those with a low CFR (111.6 and 48.8), were well above the cut-offs found in studies that were used to predict a poor prognosis (92–98 for GNI and 44.8 for PNI) [23,24]. As malnutrition was not severe in the study population, it remains uncertain whether severe malnutrition and cardiac cachexia could affect CFR in some DCM patients. For the remaining majority of DCM patients in whom malnutrition is either nonexistent or less than severe, it can be presumed that nutritional status per se does not have a major effect on CMD, based on the present findings.

In contrast to other nutritional indices, CAR was associated with CFR; this finding remained valid after adjusting for CRP and albumin, and patients with a CAR > 0.8 were approximately 13 times more likely to have a CFR < 2.0. This finding could imply that coexistence of inflammation and malnutrition induces coronary microvascular dysfunction in DCM patients, similar to malnutrition, inflammation, and atherosclerosis (MIA) syndrome seen in patients with CKD [11]. As mentioned before, other indices of malnutrition do not suggest that malnutrition was prevalent in the subset of patients with a low CFR, thus calling for other potential explanations. Since albumin is a negative acute-phase reactant, the patients with high CAR may simply represent a subset of patients with more pronounced inflammatory activation. In a previous study, patients with DCM and a CFR < 2.0 had higher serum CRP, tumor necrosis factor-α, and interleukin-1 levels compared to those with a CFR ≥ 2.0, thus supporting this latter explanation [25]. That said, our findings do not support a linear relationship between albumin and CRP (data not shown), thus suggesting that the relationship is either nonlinear or that other factors besides inflammation have a significant impact on serum albumin level in the study cohort. Finally, a low CFR in the setting of inflammation and hypoalbuminemia may imply widespread endothelial dysfunction involving both coronary and renal microvasculature, where hypoalbuminemia is a manifestation of renal dysfunction rather than malnutrition. In patients with systemic hypertension and normal coronary arteries, Tsiachris et al. have demonstrated that CFR was correlated with urinary albumin excretion, thus implying a generalized vascular disease that involves both coronary and renal vasculature in this patient group (26). While there is no direct evidence to support that a similar mechanism is operational in DCM, such a mechanism is plausible given that CMD frequently involves both organs, and albuminuria with or without manifest renal dysfunction is seen in up to 30% of patients with renal failure. Unfortunately, data from the present study are insufficient to prove or refute any of these possible explanations.

### 5.2. Possible clinical implications

Regardless of the underlying mechanism, the present findings showed that high CAR is associated with a low CFR, a condition that is linked to a worse prognosis in DCM patients [4]. While there are no data to suggest that CAR itself is associated with a poor prognosis, this relationship with CFR, as well as the prognostic implications of inflammation and hypoalbuminemia in patients with HF, could be considered as indirect evidence linking CAR with overall prognosis. Our findings also indicate that CAR has a high diagnostic accuracy in determining a CFR < 2.0; as such, a high CAR could be considered a surrogate marker for microvascular dysfunction in patients with DCM. Microvascular dysfunction is a potential target for HF, but measuring CFR or other surrogate markers of microvascular dysfunction in everyday practice remains challenging as complex or invasive methods are needed to determine microvascular dysfunction [27,28]. As such, CAR could be useful for screening patients with DCM and possible microvascular dysfunction to select patients for more definitive testing. Finally, several ongoing studies are aimed at correcting individual components of CAR—namely inflammation and hypoalbuminemia—to see whether such strategies can correct endothelial function (25). CAR could be a useful tool to monitor responses to such therapies; it could be expected that a decline in CAR would be indicative of better microvascular or endothelial dysfunction. Since the present study is not oriented towards possible clinical applications of CAR, assertions provided in this section should be considered hypothetical until more solid evidence is available.

As the present findings imply that microvascular dysfunction is related with both nutrition and inflammation, it would be reasonable to expect that strategies or treatments aimed to improve nutrition or mitigate inflammation could attenuate microvascular dysfunction. Drugs affecting the renin–angiotensin–aldosterone axis, such as ACE inhibitors, angiotensin receptor blockers, or mineralocorticoid receptor blockers, are used as the primary means of treatment in patients with heart failure and reduced ejection fraction, including those with DCM. While these drugs modify a multitude of pathways, some of their beneficial effects are exerted via blocking the proinflammatory effects of angiotensin and aldosterone [29,30]. As such, these drugs may also improve microvascular function in DCM patients and perhaps exert some of their beneficial effects via this mechanism. Indeed, evidence is available to suggest that renin–angiotensin–aldosterone system blockers improve coronary microvascular function in patients with hypertension or Syndrome X, though such evidence is lacking for DCM [31,32]. Finally, a recently published CANTOS study found that canakinumab, an interleukin-1B inhibitor that is used for the treatment of rheumatoid arthritis, can reduce hospitalizations for heart failure and that some of its effects can be mediated via improvement of microvascular function, but more data is required to make more definitive comments on this subject [33].

## 6. Study limitations

The study was done using data from a single center, the sample size was small, and the data was collected retrospectively, all limiting the quality of data used to obtain results. All findings should be interpreted in this context. That said, the data was acquired from a prospective institutional registry with an objective to study coronary flow dynamics in patients with various conditions and therefore a standard approach was used to collect data, thus mitigating the effects of the latter limitation on data quality. The results were only valid for those with nonischemic DCM and should not be generalized to all patients with HF. In each standard approach, CFR was measured only from the left anterior descending artery and therefore only represents microvascular function in a limited left ventricular territory. Nonetheless, the effects of processes that lead to nonischemic DCM are global and it can be assumed that the results could be extrapolated to other LV territories. Finally, adjustments were done for a limited number of variables due to sample size and lack of data on all variables; therefore, other factors that might act as confounders were omitted. These factors should be taken into consideration when interpreting the present findings.

## 7. Conclusion

In patients with nonischemic DCM, not malnutrition per se but the coexistence of inflammation and hypoalbuminemia, manifested as an elevated CAR, is associated with coronary microvascular function. This association remained valid after adjusting for potential confounders. Further work is required to externally validate CAR as a surrogate marker of CFR in DCM patients and to assess whether these findings are also valid for other patient groups, such as those with ischemic DCM or HF with preserved ejection fraction.
